# Protocol for a realist review of workplace learning in postgraduate medical education and training

**DOI:** 10.1186/s13643-017-0415-9

**Published:** 2017-01-19

**Authors:** Anel Wiese, Caroline Kilty, Colm Bergin, Patrick Flood, Na Fu, Mary Horgan, Agnes Higgins, Bridget Maher, Grainne O’Kane, Lucia Prihodova, Dubhfeasa Slattery, Deirdre Bennett

**Affiliations:** 1 Medical Education Unit, School of Medicine, University College Cork, Cork, Ireland; 2 Trinity College Dublin, Dublin, Ireland; 3 Dublin City University Business School, Dublin, Ireland; 4 Trinity College Dublin Business School, Dublin, Ireland; 5 School of Nursing and Midwifery, Trinity College Dublin, Dublin, Ireland; 6 Royal College of Physicians of Ireland, Dublin, Ireland; 7 Children’s University Hospital, Temple St., Dublin, Ireland

**Keywords:** Realist review, Realist synthesis, Postgraduate medical education, Graduate medical education, Workplace learning, Clinical learning environment

## Abstract

**Background:**

Postgraduate medical education and training (PGMET) is a complex social process which happens predominantly during the delivery of patient care. The clinical learning environment (CLE), the context for PGMET, shapes the development of the doctors who learn and work within it, ultimately impacting the quality and safety of patient care. Clinical workplaces are complex, dynamic systems in which learning emerges from non-linear interactions within a network of related factors and activities. Those tasked with the design and delivery of postgraduate medical education and training need to understand the relationship between the processes of medical workplace learning and these contextual elements in order to optimise conditions for learning. We propose to conduct a realist synthesis of the literature to address the overarching questions; how, why and in what circumstances do doctors learn in clinical environments? This review is part of a funded projected with the overall aim of producing guidelines and recommendations for the design of high quality clinical learning environments for postgraduate medical education and training.

**Methods:**

We have chosen realist synthesis as a methodology because of its suitability for researching complexity and producing answers useful to policymakers and practitioners. This realist synthesis will follow the steps and procedures outlined by Wong et al. in the RAMESES Publication Standards for Realist Synthesis and the Realist Synthesis RAMESES Training Materials. The core research team is a multi-disciplinary group of researchers, clinicians and health professions educators. The wider research group includes experts in organisational behaviour and human resources management as well as the key stakeholders; doctors in training, patient representatives and providers of PGMET.

**Discussion:**

This study will draw from the published literature and programme, and substantive, theories of workplace learning, to describe context, mechanism and outcome configurations for PGMET. This information will be useful to policymakers and practitioners in PGMET, who will be able to apply our findings within their own contexts. Improving the quality of clinical learning environments can improve the performance, humanism and wellbeing of learners and improve the quality and safety of patient care.

**Systematic review registration:**

The review is not registered with the PROSPERO International Prospective Register of Systematic Reviews as the review objectives relate solely to education outcomes.

**Electronic supplementary material:**

The online version of this article (doi:10.1186/s13643-017-0415-9) contains supplementary material, which is available to authorized users.

## Background

Postgraduate medical education and training (PGMET) is a complex social process which happens predominantly during the delivery of patient care. The social, cultural and material context for PGMET is the clinical learning environment (CLE) [1] which has been described as ‘*the foundation of graduate medical education’* [2]. There is evidence that learners and learning are shaped by clinical learning environments; their performance [3–7], humanism [8, 9] and psychological health [10–13] are supported in high quality environments. Supportive clinical learning environments can contribute to better patient care through these direct effects on residents, and their current practice. Empirical research has also shown that the hidden and informal curricula of CLEs shape the future practice of residents [14, 15]. It has also been recognised that environments which lead to good patient outcomes are also positive learning environments [16, 17]. These are important drivers to understand how and why doctors’ learning is supported in clinical environments.

### Complexity in clinical environments

Health care systems internationally are under strain, facing increasing demands with limited resources [18, 19]. Although patients have shorter hospital stays, inpatients are sicker and healthcare has become more complex and expensive [20]. Short staffing and overcrowding are often features of the environments in which doctors learn [21]. Capping of working hours for doctors-in-training, driven by concerns for doctor wellbeing and patient safety, has resulted in a move to shift work and fewer total hours worked, raising concerns about unintended consequences for learning and delivery of care [22]. Clinical workplaces are complex, dynamic systems in which learning emerges from non-linear interactions within a network of related factors and activities [23, 24]. The key components of CLEs include appropriate opportunities to learn through practice, supervision, assessment and feedback, social support in relationships with consultants, peers, nurses and allied healthcare professionals, working hours and conditions, and resources [25, 26]. Delivery of patient care, adherence to working hours legislation, focus on patient safety and resource management are activities which may compete with, as well as generate, learning in clinical workplaces. Learning, clinical environment and working conditions are closely intertwined. Working under poor conditions is linked to trainee stress and burnout, which may impact learning, humanism and professional identity formation [11, 27]. These factors may impact each other in unanticipated ways with unintended consequences. Those tasked with the design and delivery of postgraduate medical education and training need to understand the relationship between the processes of medical workplace learning and these contextual elements in order to optimise conditions for learning. This review aims to produce a detailed description of these relationships grounded in the literature and workplace learning theory.

### Researching complexity to inform policy and practice

Researching how learning happens in clinical environments and how we can support it requires the use of an approach which recognises the complexity of the environment and of postgraduate training itself. We have been funded by the Health Research Board (Ireland), the Medical Council of Ireland and the Irish Health Service Executive (National Doctors Training and Planning) to conduct a project comprised of three studies triangulating on the ways in which clinical learning environments shape postgraduate trainees’ learning. Our funders have not been involved in the development of the protocol. Our overarching aim is to produce guidelines and recommendations for the design of high quality clinical learning environments for postgraduate medical education and training. As part of this project, we propose to conduct a realist synthesis of the literature to explore the overarching questions; how, why and in what circumstances do doctors learn in clinical environments? Realist review will build and refine a theory of postgraduate medical education and training to address these questions.

## Methodology

We have chosen realist synthesis as a methodology because of its suitability for researching complexity and producing answers useful to policymakers and practitioners.

Specific research questions are the following:

1. What are the mechanisms by which postgraduate workplace learning results in its intended outcomes?
2. What are the important contexts which determine whether the different mechanisms produce their intended outcomes?
3. In what circumstances is postgraduate medical workplace learning effective?

Realist review is an interpretative theory-driven narrative summary of the literature describing how, why and in what circumstances complex social interventions work. A complex intervention is one whose outcome is dependent on the interaction between its participants and their context; in this case, doctors in training and the clinical learning environment. Complex interventions ‘often have multiple components (which interact in non-linear ways) and outcomes (some intended and some not) and long pathways to the desired outcome(s)’ [28]. Traditional systematic reviews of such interventions tend to have mixed results and do not explain how or why the intervention worked. They generally try to eliminate the effect of context rather than understand its impact. Realist review addresses these shortcomings by producing rich contextual information which policymakers and practitioners can apply to their own circumstances [29].

Realist philosophy holds that outcomes of an intervention are not deterministic or simply linear but are context dependent. Realist synthesis translates the findings of empirical studies into context, mechanism and outcome (CMO) configurations, which state that in a certain context a particular mechanism generates a particular outcome. Social mechanisms refer to the ‘underlying entities, processes or (social) structures which operate in particular contexts to generate outcomes of interest’ [30]. An intervention may be comprised of multiple mechanisms both planned and unintentional. Identification of CMO configurations is informed by programme theory, or the underlying assumptions of how the intervention is supposed to work, and relevant middle range theories, in this case, theories of workplace learning. Using theory to identify CMO configurations focuses reviewers on the underlying and transferable aspects of programmes described rather than on their specific minutiae [30]. Realist review seeks to identify ‘demi-regularities’ within the complexity of interventions, based on the expectation that although outcomes will vary in different contexts, that there will be some patterning in CMO configurations [31]. Theory is also generated, tested and refined through this process.

The core research team is a multi-disciplinary group of researchers, clinicians and health professions educators. The wider research group includes the key stakeholders; doctors in training, patient representatives and the Royal College of Physicians of Ireland, the largest postgraduate training body in Ireland, with responsibility for almost half of all postgraduate trainees nationally. The core research team will also undertake training and ongoing consultation with methodological experts during the course of the study.

## Procedures

This realist synthesis will follow the steps and procedures outlined RAMESES Publication Standards for Realist Synthesis [28] and associated training materials [30] in an iterative manner. Realist principles will be embedded in all stages of the review process. A PRISMA-P checklist has been completed and is available as an additional file (see Additional file 1).

### Defining the scope of the review

Clinical learning environments in postgraduate medical education and training is a broad topic. Initial rough searches of the literature suggest that there is a substantial published literature in this area. Focussing the review question will be an iterative process consisting of exploration of the literature and relevant programme theories as well as consultation with experts and stakeholders. We envisage that focussing of the review will be guided by the evidence as it is discovered and the need to ensure a manageable volume of literature for synthesis.

We will identify programme and substantive workplace learning theories which will support the identification of the key areas on which to focus and the most relevant literature to consider. There are several theories of learning which can inform our understanding of the processes and outcomes of doctors’ workplace learning and how clinical environments might impact these. Communities of practice theory [32] emphasises the importance of participation in practice and connection, recognition and belonging within a community. Cognitive apprenticeship theory [33, 34] provides an account of how people learn from each other, through observation, modelling and reflection. The frameworks for workplace learning developed by Billett [35] and Teunissen [36] may also prove relevant to this review. An initial programme theory for workplace learning in postgraduate medical education and training will be developed and expressed in realist terms. This theory will be refined as the review progresses and becomes more focussed.

Regular meetings will be held with the wider project team to discuss and define the key aspects of the review, to ensure consensus on review focus. Additionally, the scope of the review will be informed by another study into clinical learning environment being undertaken by the group. This is a consensus building study which will use group concept mapping to capture the perspectives of multiple stakeholders such as trainers, trainees, allied health professionals and hospital management on priority areas for improvement within clinical learning environments. The findings of this study will help to direct the focus of the realist synthesis.

### Search strategy

Unlike the search strategy of traditional systematic reviews, searches in realist synthesis do not aim to uncover every published paper addressing the topic, but rather to balance comprehensiveness with theoretical saturation. Searching is iterative and as synthesis and theory refinement occur further searches may be necessary to test the emerging theory. Initially, we will perform an electronic search in the following databases: Academic Search Complete, Australian Education Index, British Education Index, CINAHL, ERIC, MEDLINE, PsycInfo and SocIndex. Search terms will be developed and trialled iteratively, and in discussion within the wider research team and a librarian. Both MeSH (medical subject headings) and free text will be employed to ensure breadth and depth of coverage. The search will be adjusted as required in each database. We will supplement searches by reviewing the reference lists of included studies and relevant review articles. We will also search the following journals by hand: *Academic Medicine*, *Advances in Health Sciences*, *Graduate Medical Journal*, *Medical Education*, *Medical Teacher and Postgraduate Medical Journal*. We will check the validity of our search by contacting experts in the field of workplace learning to identify the key papers. Additional rounds of searching may be added throughout the review process to further explore particular areas of interest. Searching will cease when sufficient evidence has been found to demonstrate the coherence and plausibility of the refined programme theory.

Our core inclusion criteria will be (1) papers related to postgraduate medical education and training in the clinical setting, (2) quantitative, qualitative and mixed-method studies, (3) papers published in English and (4) papers published between 1995 and 2016. Exclusion criteria include (1) non-empirical papers including commentaries, letters, editorials and reviews, (2) papers related to undergraduate medical education and (3) research on simulation or other non-clinical interventions.

### Study selection criteria

Titles and abstracts will be imported into EndNote and screened by the core research team using the criteria outlined above. Full texts of articles deemed potentially relevant will be retrieved and evaluated for inclusion in the data extraction stage. Inclusion and exclusion decisions will be based on whether the findings can contribute to theory testing and refinement and whether the methods used to generate findings are credible and trustworthy.

The questions that will guide us selecting papers based on relevance are the following: Does the study relate directly to the clinical learning environment of medical trainees? Is the study rich enough in information on context, mechanism and outcomes? These questions will ensure that the sources identified allow the team to make sense of the subject area, in order to develop, refine and test theories, and to support inferences made about mechanisms. Reasons for exclusion of papers will be thoroughly documented to ensure transparency. Searching will be iterative and will discontinue when sufficient evidence is found that it is reasonable to claim that the programme theory is coherent and plausible.

Quality of the papers selected for data extraction will be assessed, and methodological limitations will be identified and taken into account during the data synthesis phase. Realist synthesis is an interpretative approach to the literature and the RAMESES Realist Synthesis Training Materials do not recommend using a strict checklist approach to quality, as this can lead to exclusion of relevant papers early in the process. Our assessment of quality will involve the use of checklists, for example CASP, as sensitising influences, but will lean towards inclusion of data from relevant studies with some methodological shortcomings.

### Data extraction

In realist review, data extraction may include descriptions and explanations of how and why the programme theory may have worked in particular contexts [28]. We will use two approaches to extract data from selected studies. Firstly, an electronic data extraction sheet will be used in order to record study identification details (authors, title, publication, etc.), geographical area in which the study was conducted, specific population and methodology used. Comments on the rigour and trustworthiness of the study will also be included. Secondly, we will import the selected papers into NVivo and code the results and discussions sections in order to identify context, mechanism, outcome configurations in the findings. Three members of the research team will undertake data extraction, and cross checking will be undertaken to identify any inconsistencies, inaccuracies or oversights. Any discrepancies will be discussed and resolved among the core research team with reference to the wider research team if necessary.

### Data analysis and synthesis

Analysis and synthesis will proceed in NVivo as we identify recurring relationships between contexts, mechanisms and outcomes in the selected papers. This process will be guided by programme and substantive theories. We will look for predictable patterns (demi-regularities) to determine how similar mechanisms act in different contexts to generate outcomes. Emerging findings will be challenged and contrary examples will be sought in the data and in theory. This process will allow information on outcomes that differ in comparable circumstances, for contradictory outcomes to occur in particular contexts, and for judgements of the strength/weaknesses of research methods to be integrated into the synthesis. These findings will feedback into theory refinement. The following conceptual tools will be applied during this phase [30];

- **juxtaposing** (“for instance, when one study provides the process data to make sense of the outcome pattern noted in another”)
- **reconciling** (identifying differences which explain apparently contradictory sets of findings)
- **adjudicating** between studies (based on the quality of research);
- **consolidating** (building ‘multi‐faceted explanations of success’)
- **situating** (‘this mechanism in context A, that one in context B”)

We will adopt an iterative and explanatory approach to synthesis of the data. The findings will be synthesised to be of practical use and will be reported according to the RAMESES reporting standards for realist syntheses [28].

## Discussion

This study will draw from the published literature and programme and substantive theories of workplace learning, to describe context, mechanism and outcome configurations for PGMET. Realist synthesis methodology is appropriate to explore a complex intervention such as PGMET, which takes place in complex learning environments, in which learning is not the primary activity. By identifying causal mechanisms in PGMET, it may be possible to design clinical learning environments that are effective for learning, and create satisfactory working conditions for doctors in training. The results of this realist synthesis will be useful to policymakers and practitioners in PGMET, who will be able to apply the findings within their own contexts. Improving the quality of clinical learning environments can improve the performance, humanism and wellbeing of learners and ultimately the quality and safety of patient care.

## Additional file

Additional file 1: PRISMA-P checklist realist review: this is the completed PRISMA-P checklist for the review protocol. (DOCX 29 kb)
